# Automated feature quantification of Lipiodol as imaging biomarker to predict therapeutic efficacy of conventional transarterial chemoembolization of liver cancer

**DOI:** 10.1038/s41598-020-75120-7

**Published:** 2020-10-22

**Authors:** Sophie Stark, Clinton Wang, Lynn Jeanette Savic, Brian Letzen, Isabel Schobert, Milena Miszczuk, Nikitha Murali, Paula Oestmann, Bernhard Gebauer, MingDe Lin, James Duncan, Todd Schlachter, Julius Chapiro

**Affiliations:** 1 Department of Radiology and Biomedical Imaging, Yale School of Medicine, 333 Cedar Street, New Haven, CT 06510 USA; 2 Berlin Institute of Health, Institute of Radiology, Charité - Universitätsmedizin Berlin, Corporate Member of Freie Universität Berlin, Humboldt-Universität, 10117, Berlin, Germany; 3 Department of Biomedical Engineering, Yale School of Engineering and Applied Science, New Haven, CT 06520 USA; 4 Faculty of Medicine, Albert-Ludwigs-University, Freiburg, Germany; 5 Faculty of Medicine, Heinrich-Heine-University, Düsseldorf, Germany

**Keywords:** Predictive markers, Translational research

## Abstract

Conventional transarterial chemoembolization (cTACE) is a guideline-approved image-guided therapy option for liver cancer using the radiopaque drug-carrier and micro-embolic agent Lipiodol, which has been previously established as an imaging biomarker for tumor response. To establish automated quantitative and pattern-based image analysis techniques of Lipiodol deposition on 24 h post-cTACE CT as biomarker for treatment response. The density of Lipiodol deposits in 65 liver lesions was automatically quantified using Hounsfield Unit thresholds. Lipiodol deposition within the tumor was automatically assessed for patterns including homogeneity, sparsity, rim, and peripheral deposition. Lipiodol deposition was correlated with enhancing tumor volume (ETV) on baseline and follow-up MRI. ETV on baseline MRI strongly correlated with Lipiodol deposition on 24 h CT (*p* < 0.0001), with 8.22% ± 14.59 more Lipiodol in viable than necrotic tumor areas. On follow-up, tumor regions with Lipiodol showed higher rates of ETV reduction than areas without Lipiodol (*p* = 0.0475) and increasing densities of Lipiodol enhanced this effect. Also, homogeneous (*p* = 0.0006), non-sparse (*p* < 0.0001), rim deposition within sparse tumors (*p* = 0.045), and peripheral deposition (*p* < 0.0001) of Lipiodol showed improved response. This technical innovation study showed that an automated threshold-based volumetric feature characterization of Lipiodol deposits is feasible and enables practical use of Lipiodol as imaging biomarker for therapeutic efficacy after cTACE.

## Introduction

Despite advances in diagnosis and therapy, liver cancer—both primary and metastatic—remains one of the leading causes of cancer-related death worldwide and incidence rates continue to rise^1–3^. As only a minority of patients are diagnosed at early and thus curative stages, palliative image-guided intraarterial therapies represent key therapeutic options, particularly for medium to advanced stage Barcelona clinic liver cancer (BCLC) B and C stage hepatocellular carcinoma (HCC). Among those, conventional transarterial chemoembolization (cTACE) is the most commonly used, with level 1A evidence available in patients with HCC^4,5^. But cTACE is also the most commonly used intraarterial therapy in unresectable intrahepatic cholangiocarcinoma (ICC) and neuroendocrine tumor metastases^6,7^. In cTACE, the oily contrast medium Lipiodol is emulsified with chemotherapeutic drugs and administered into tumor feeding branches of the hepatic artery^8^. Lipiodol plays a unique multifunctional role in cTACE: in addition to drug delivery capabilities with transient microembolic effects, its radiopacity allows drug delivery visualization on post-procedural CT, making it a promising imaging biomarker^9^.

Previous studies suggest that Lipiodol retention in the tumor is strongly correlated with tumor necrosis on follow-up imaging and histopathology^10–13^. Additionally, baseline imaging features, particularly those indicative of tumor vascularity, such as arterial enhancement, have been previously thought to predict tumor response to cTACE^14,15^. The dual blood supply of the liver and the complex microvascular architecture of hepatic malignancies with shunts between the hepatic artery and portal vein suggest that not only the coverage, but also density and spatial distribution of Lipiodol deposition in the tumor may correlate with therapeutic efficacy^9,16,17^. However, clinical implications of varying Lipiodol density and its spatial distribution on imaging have not yet been studied and their impact on tumor response remains unclear. Previous studies quantified Lipiodol deposition using manual segmentations or semi-automatic threshold-based techniques that were coarse, reader-dependent and potentially prone to bias given significant pattern heterogeneity and radiomic complexity of Lipiodol, limiting their effectiveness as predictable imaging biomarkers^10,11,18^. Translating Lipiodol deposition on imaging into a reliable surrogate marker for tumor response requires robust automated techniques that provide efficient, standardized methods for analyzing and describing its appearance on CT.

In light of the unmet clinical need and given the aforementioned multifunctional properties of Lipiodol, the purpose of this study was to establish automated quantification instruments to allow practical deployment of Lipiodol as a predictive imaging biomarker for therapeutic efficacy on 24 h postprocedural CT that is commonly used to verify Lipidol deposition after cTACE^19–21^.

## Materials and methods

### Patient/tumor selection

A total of 42 patients with primary and secondary liver cancer treated using cTACE (2012–2018) according to prospective clinical trial protocols (NCT01877187, NCT02753881) were identified by a multi-disciplinary team for secondary retrospective data analysis. The study was conducted in accordance with the Declaration of Helsinki on Ethical Principles for Medical Research Involving Human Subjects and approved by the Yale institutional review board (Yale Human Research Protection Program). Written informed consent was obtained from all patients. Inclusion criteria were the presence of radiologically confirmed HCC or other solid liver tumors (intrahepatic cholangiocarcinoma (ICC) or liver-predominant metastatic disease), age ≥ 18, Eastern Cooperative Oncology Group (ECOG) performance status 0–2, Child Pugh class A or B (up to 9). Other main exclusion criteria were any contraindication to doxorubicin or mytomycin c, severe cardiac or systemic disease, known allergy to Lipiodol, poppy seed oil or iodinated contrast agent, main portal vein thrombosis and patients who were pregnant or breastfeeding. For our retrospective analysis all patients had to have an MRI before and after cTACE, as well as a 24 h post-procedural non-contrast CT of the abdomen according to standardized protocols. The average time interval between the baseline MRI and the 24 h CT was 16.0 ± 14.8 days [1–52 days] (mean ± SD, [range]) and the average time interval between the 24 h CT and the follow up MRI was 29.2 ± 6.0 days [20–47 days].

Up to five treated lesions were analyzed for each patient. Atypical appearing lesions (e.g. presumed HCC lesions not meeting Liver Imaging Reporting and Data System (Li-RADS) criteria), non-target and previously treated lesions were excluded. Only lesions with a diameter ≥ 1 cm were included. The final study cohort included 42 patients with 65 lesions.

### TACE procedure

The cTACE procedure was performed according to the institutional review board approved protocols. In multiple angiographic steps the tumor-feeding vasculature was identified. Patients underwent lobar or selective cTACE. Lipiodol (10 cc) was mixed with 50 mg doxorubicin and 10 mg of mitomycin c to create an emulsion. The water-in-oil emulsion was mixed thoroughly using a push-and-pull method to obtain a homogenous satin red solution with high stability^19^. Note that in the vast majority of cases, especially hypervascular tumors, the ratio of Lipiodol to chemotherapy was slightly greater than 1:1 in order to create a true water-in-oil emulsion. The procedure was started with a 1.5:1 ratio and depending on arterial flow was either continued at that same ratio or decreased slightly if the flow became too sluggish to accommodate the higher viscosity of the emulsion. The exact amount of chemoembolization material administered was titrated to the area being treated. Embolization was achieved by administration of about one vial of Embospheres (100–300 µm, Merit Medical). Delivery of the entire dose of chemotherapy was considered the technical endpoint whereas arterial flow reduction (2–5 heart beats to clear the contrast column) was considered the angiographic endpoint. All procedures were performed by board-certified interventional radiologists who had between 7 and 20 years of experience.

### CT/MRI acquisition

MRI was performed on a 1.5 or 3-T scanner (Magnetom Avanto or Skyra, Siemens Medical Solutions). The protocol included anatomical and contrast-enhanced T1-weighted sequences with arterial, portal venous and delayed phase (20 s, 70 s and 3 min after contrast administration) using a gadolinium-based contrast agent. Additionally, all patients received a non-contrast abdominal CT 24 h after cTACE to visualize Lipiodol deposition.

### Tumor segmentation and MR image analysis

**Figure 1:**
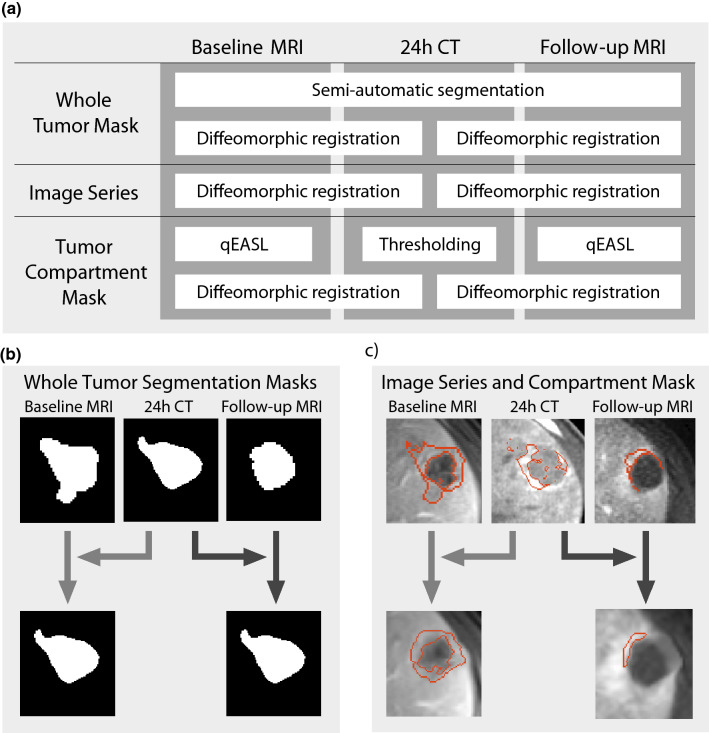
Segmentation and registration workflow. (**a**) Tumors were semi-automatically segmented on all three imaging time points (baseline MRI, 24 h CT, follow-up MRI). Compartment masks on MRI (viable/necrotic tumors masks) were produced using quantitative European Association for the Study of the Liver (qEASL), while compartment masks on 24 h CT (low/mid/high density Lipiodol masks) were generated using Hounsfield Unit thresholds. (**b**) Each of the two MRI tumor segmentation masks were diffeomorphically registered to the 24 h CT tumor mask. These two registration procedures (represented by arrows of differing shades of gray) each generated a separate transformation matrix. (**c**) This transformation matrix was used as a template to simultaneously register the corresponding images and compartment masks to 24 h CT.

A semi-automatic tumor segmentation software (IntelliSpace Portal Version 8, Philips ICAP) was used to segment target lesions in 3D at all imaging timepoints. As shown in Fig. 1, these segmentation masks of the tumor were used to register baseline MRI, 24 h CT and follow-up MRI. For this step, the Diffeomorphic “Demons” Registration algorithm was used, as implemented in the Insight Segmentation and Registration Toolkit (The Insight Software Consortium, https://www.itk.org)^23^. Diffeomorphic registration finds a smooth mapping between the points in the binary masks, yielding a transformation that can be applied to the raw images. It is a non-linear technique that performs approximate second-order minimization of a “correspondence energy” that incorporates an intensity matching term (how well aligned the fixed and moving images are) and a regularization term (to penalize large deformations)^24^. The image transformation was derived from the tumor segmentation masks rather than the raw images, as the latter would bias other analyses by favoring overlap between voxels of high intensity (enhancing tumor on MRI and Lipiodol deposition on CT).

On baseline and follow-up MRI, a 1 cm^3^ volume of interest (VOI) was placed into the background liver parenchyma in the ipsilateral lobe of the dominant lesion outside the treatment zone on arterial phase. The average signal intensity was used as a threshold to separate regions of non-enhancing, presumably necrotic from enhancing, presumably viable tissue. Viable tumor on MRI (enhancing tumor volume (ETV)) was defined as voxels where enhancement is ≥ 2 standard deviations greater than the enhancement of the VOI (quantitative European Association for the Study of the Liver (qEASL) approach using IntelliSpace Portal V8, Philips ICAP)^25,26^. Overall reduction in viable tumor tissue was quantified as the percentage decrease in viable tumor volume between baseline and follow-up MRI. To assess radiographic tumor response of individual lesions the qEASL criteria were used because enhancement based response criteria have be shown to predict tumor necrosis after cTACE most accurate^25,27–29^. According to qEASL, lesions were considered responders if they had a reduction of enhancing tumor volume of at least 65%. The percent reduction in the viable tumor areas was calculated as the number of voxels in the region that changed from arterially hyperenhancing to non-enhancing on MRI, taken as a percentage of the total number of arterially hyperenhancing voxels on baseline MRI.

### CT image analysis and post-processing

#### Lipiodol density

**Figure 2:**
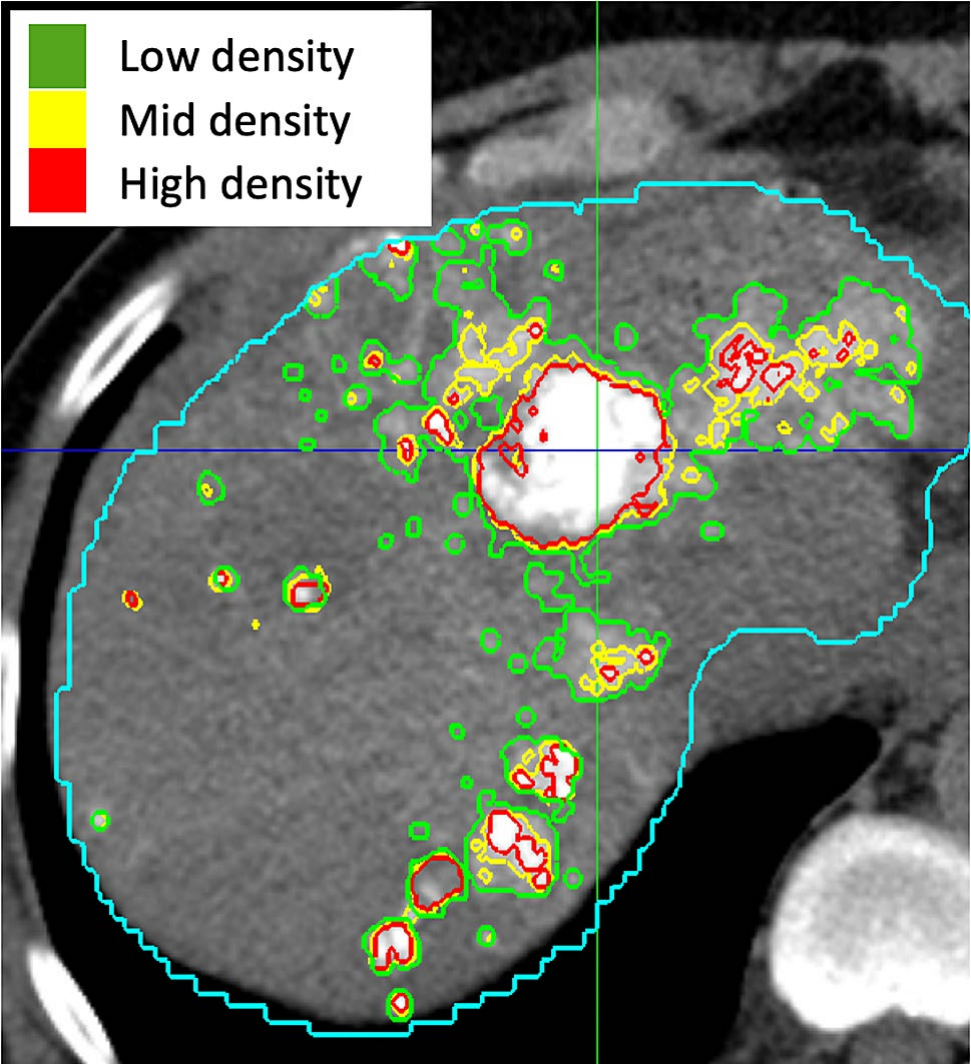
Lipiodol densities on an example CT. The presence and density of Lipiodol on 24 h post-TACE CT was automatically characterized using cut-off values of 87 HU, 155 HU, and 241 HU. The blue contour indicates the automated whole liver segmentation. This example illustrates a homogeneous pattern with some peripheral deposition.

On 24 h CT, each tumor was divided into regions with or without Lipiodol using an intensity threshold in Hounsfield units (HU). The Lipiodol coverage of a tumor region was quantified as the percentage of voxels in that region that contained Lipiodol of any density. Additionally, areas of Lipiodol deposition were further characterized according to their density as illustrated in Fig. 2. Three intensity thresholds needed to be determined to characterize the density of Lipiodol: one threshold separating no Lipiodol from low density Lipiodol, one separating low from medium density, and one separating medium from high density Lipiodol. The first intensity threshold was determined by selecting regions in the parenchyma of each 24 h CT in which no Lipiodol deposition was observed. This threshold was set as the 99th percentile of the voxel intensities in these regions. First, each tumor was individually thresholded into regions of lower and higher deposition. Two thresholding techniques were used, one based on cross entropy and one based on variance, and their average was taken^30,31^. This approach was designed to consider the narrow HU ranges of Lipiodol deposition patterns in most tumors, which means that many tumors will have no more than two distinct Lipiodol densities, making it suitable for producing only a single threshold. The 33rd percentile of thresholds across all tumors was then used as the overall threshold between low and medium density Lipiodol, and the 67th percentile was used as the threshold between medium and high density Lipiodol. These percentiles are chosen so that at least one of these two overall thresholds will provide separation between Lipiodol regions in most tumors. This technique is not biased by the size of the tumor and is not biased by outliers with very high Lipiodol deposition. Due to possible imprecision in the segmentation and registration, subregions were only analyzed if they contained at least 50 voxels.

#### Lipiodol deposition patterns

**Figure 3:**
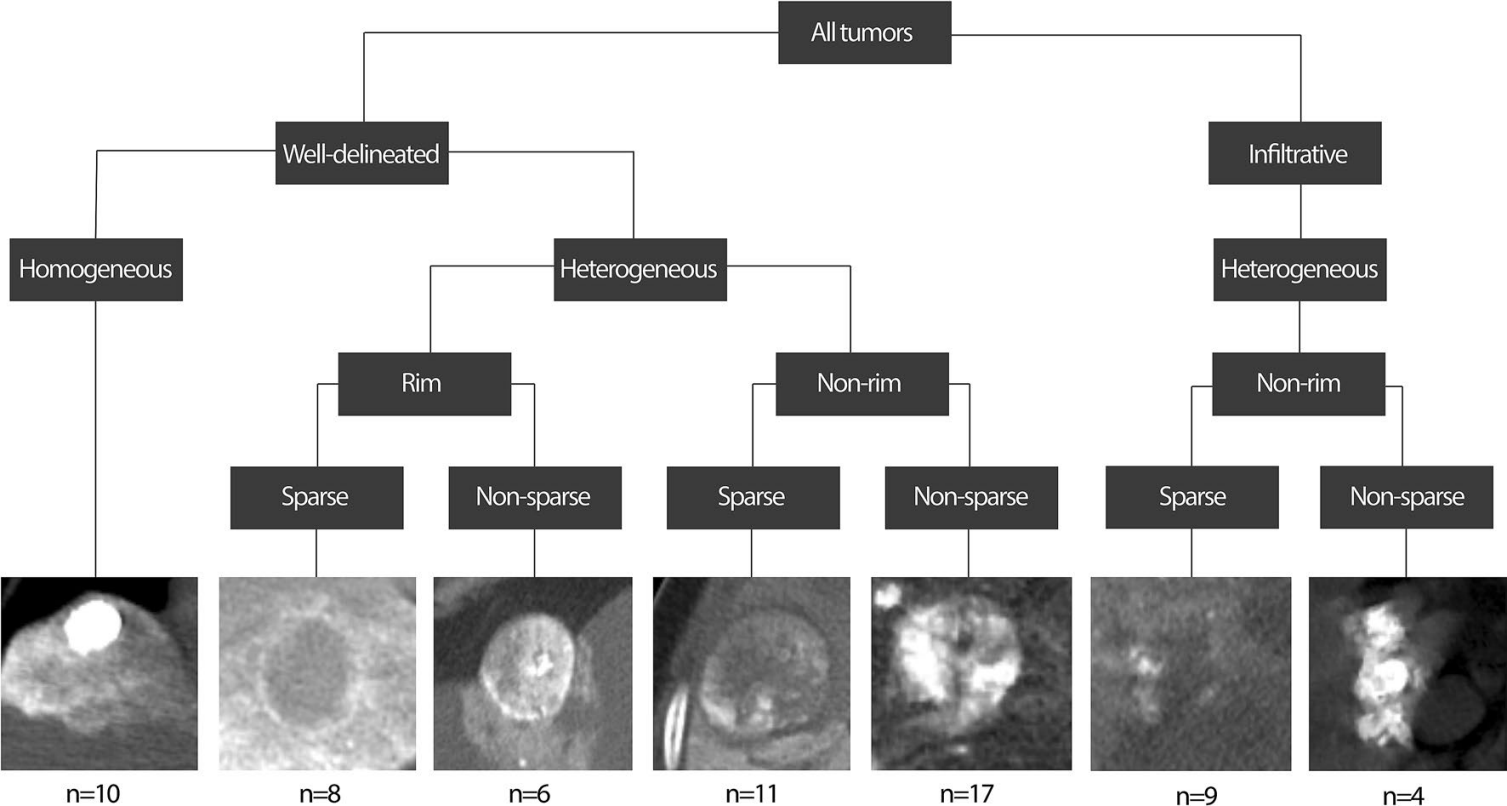
Categorization of Lipiodol deposition patterns. Well-delineated and infiltrative lesions were manually characterized. Then, each tumor’s Lipiodol deposition was automatically characterized based on its homogeneity, rim presence and sparsity. Deposition was considered homogeneous if ≥ 85% of the tumor volume contained medium or high density Lipiodol. Lipiodol deposition was described as sparse if ≤ 20% of the tumor volume had medium and ≤ 10% of the tumor volume had high density Lipiodol. Tumors not meeting the criteria for sparsity were defined as non-sparse tumors. Rim deposition required that the outer portion of the tumor had denser Lipiodol deposition than its core. (Location of the tumors: well-delineated lesions: homogeneous: segment Iva (diameter 2.0 cm), rim sparse: segment V (diameter 5.9 cm), rim non-sparse: segment IVb (diameter 4.5 cm), non-rim sparse: segment VI (diameter 4.3 cm), non-rim non-sparse: segment VIII (diameter 6.2 cm), Infiltrative lesions: sparse: segment V (diameter 6.8 cm) non-sparse: segment IVb (diameter 4.1 cm).

Each lesion’s overall Lipiodol deposition pattern was described based on the presence of three imaging features on 24 h CT: homogeneity, sparsity, and rim deposition (Fig. 3 and Table 1).

**Table 1:** Definition of Lipiodol deposition patterns. This table gives an overview over the acquisition criteria for respective Lipiodol deposition patterns on 24h CT.

Pattern	Definition
Homogeneous	≥ 85% of the tumor contains medium or high density Lipiodol
Sparse	≤ 20% of the tumor contains medium density Lipiodol and ≤ 10% high density Lipiodol. Tumors not meeting the criteria for sparsity were defined as non-sparse tumors
Rim	The outer portion of the tumor has denser Lipiodol deposition than its core
Peripheral	Percentage of the tumor surface area that is directly exposed to peritumoral Lipiodol of any density

##### Homogeneous deposition

Deposition was considered homogeneous if ≥ 85% of the tumor volume contained medium or high density Lipiodol. This corresponds to bright tumors that are mostly or completely filled.

##### Sparse deposition

Lipiodol deposition was described as sparse if ≤ 20% of the tumor volume had medium and ≤ 10% of the tumor volume had high density Lipiodol. At the opposite end from homogeneous deposition, sparse deposition corresponds to tumors that are mostly dark and unfilled. Tumors not meeting the criteria for sparsity were defined as non-sparse tumors.

##### Rim deposition

Rim deposition required that the outer portion of the tumor had denser Lipiodol deposition than its core. To isolate the rim of a tumor, a series of image processing operations were applied to the tumor segmentation mask. First, the volume of the tumor was determined and the radius of a sphere with the same volume was calculated. Morphological erosion was applied to the mask, removing any voxels that are within a certain distance from the surface of the tumor. This distance was set to 15% of the radius found above. The mask obtained in this way was considered to be the core of the tumor. By subtracting the original tumor mask by the mask of its core, a mask was obtained for the rim of the tumor. A base intensity value was established as the maximum of 87 HU (low Lipiodol density threshold) and the average intensity of the core of the tumor. The amount of rim deposition in each tumor was quantified as the average amount by which voxels in the rim exceeded this base intensity value, where voxels that did not exceed the base intensity contributed 0 to this mean. The cut-off for describing a tumor as rim-depositing was determined empirically as 17 HU above the base intensity.

##### Peripheral deposition

In addition to characterizing Lipiodol deposition within a tumor, peritumoral deposition was also quantified as the percentage of the tumor surface area that is directly exposed to peritumoral Lipiodol of any density. To isolate the periphery of a tumor, another series of image processing operations were applied to the tumor segmentation mask. Morphological dilation was applied to the mask, adding any voxels within 3.5 mm of the surface of the tumor. 3.5 mm was selected as it was large enough to prevent image noise and small errors in the tumor mask from dominating this space, and small enough that it would most likely be dominated by Lipiodol that was drained from the tumor vessels rather than off-target Lipiodol accumulating in healthy liver parenchyma. The original tumor mask was subtracted from the dilated mask, resulting in a mask that represents the periphery of the tumor. The peripheral deposition was quantified as the percentage of voxels in this periphery mask that exceeded an intensity of 87 HU (low Lipiodol density threshold). The criteria for peripheral deposition of Lipiodol as well as the other patterns were determined empirically.

### Statistical analysis

Statistical tests were performed using GraphPad Prism 7.0. A *p* value < 0.05 was considered statistically significant. In order to correlate Lipiodol coverage with tumor entities, growth (well-delineated or infiltrative) or TACE approach (lobar or selective) Mann–Whitney U (MWU) and Kruskal–Wallis (KW) test were used. MWU and KW test were also used to correlate reduction in viable tumor tissue with the aforementioned subgroups and Lipiodol deposition patterns. The difference in Lipiodol coverage and densities between viable and necrotic tumor areas on baseline MRI was evaluated using the Wilcoxon-signed rank (WSR) test. Peripheral Lipiodol deposition was correlated with overall reduction in viable tumor tissue using linear regression and Spearman’s rank-order correlation. Details are described in supplementary materials.

## Results

### Patient/tumor selection

This study included 42 patients with a mean age of 62.2 ± 9.9 years (mean ± SD). The 65 analyzed lesions included 36 (55.4%) HCCs, 15 (23.1%) ICCs and 14 (21.5%) metastases from neuroendocrine tumors and ocular melanoma. Further characteristics of the study population are summarized in Table 2.

**Table 2:** Summary of the study population. The numerical data are summarized as mean ± standard deviation and the categorical data are shown as frequency (percentage). HBV, hepatitis B virus; HCV, hepatitis C virus; ECOG , eastern cooperative oncology group (performance status); HCC, hepatocellular carcinoma; ICC, intrahepatic cholangiocarcinoma; BCLC, barcelona clinic liver cancer; TTB, total tumor burden (TTB [%] was defined as the ratio of tumor volume and liver volume); ETB,  enhancing tumor burden (ETB [%] was defined as the ratio of enhacing tumor volume (ETV) and liver volume); TACE, transarterial chemoembolization.

**Patients (n = 42)**	
Age (years)	62.2 ± 9.9
Sex	
Male	32 (76.2%)
Female	10 (23.8%)
Ethnicity	
Caucasian	30 (71.4%)
African American	9 (21.4%)
Hispanic	1 (2.4%)
Asian/Pacific	1 (2.4%)
Others	1 (2.4%)
Cirrhosis	25 (59.5%)
Child–Pugh A	17 (40.5%)
Child–Pugh B	8 (19.0%)
HBV	3 (7.1%)
HCV	18 (42.9%)
ECOG	
0	29 (69.0%)
≥ 1	13 (31.0%)
BCLC (HCC patients only)	26 (61.9%)
A	13 (31.0%)
B	11 (26.2%)
C	2 (4.8%)
Unifocal	20 (47.6%)
Multifocal	22 (52.4%)
Tumor burden	
TTB	9.3 ± 13.1%
ETB	5.4 ± 8.9%
**Analyzed lesions (n = 65)**	
Entity	
HCC	36 (55.4%)
ICC	15 (23.1%)
Metastases	14 (21.5%)
Neuroendocrine	13 (20.0%)
Ocular Melanoma	1 (1.5%)
Baseline tumor size (cm)	4.7 ± 3.6
< 3 cm	26 (40.0%)
≥ 3 cm	39 (60.0%)
Tumor growth type	
Well-delineated	52 (80.0%)
Infiltrative	13 (20.0%)
TACE approach	
Selective	33 (50.8%)
Lobar	32 (49.2%)

### Image analysis and tumor characteristics

The minimum intensity threshold for Lipiodol was found to be 87HU. A threshold of 155HU separated low from medium Lipiodol density, and a threshold of 241HU separated medium from high Lipiodol density. The cross-entropy technique yielded a 33rd percentile of 159HU and 67th percentile of 239HU; the variance-based technique yielded thresholds of 151HU and 243HU. The average of these was taken. The classification of Lipiodol deposition patterns is shown in Fig. 3. Of the 52 well-delineated lesions, 10 were automatically identified to have homogenous, 19 sparse, and 14 rimmed Lipiodol deposition. 8 lesions with rim deposition showed sparse and 6 non-sparse Lipiodol deposition. 13 infiltrative lesions were identified, with 9 lesions showing sparse and 4 showing non-sparse Lipiodol deposition.

### Baseline tumor enhancement and Lipiodol deposition

**Figure 4:**
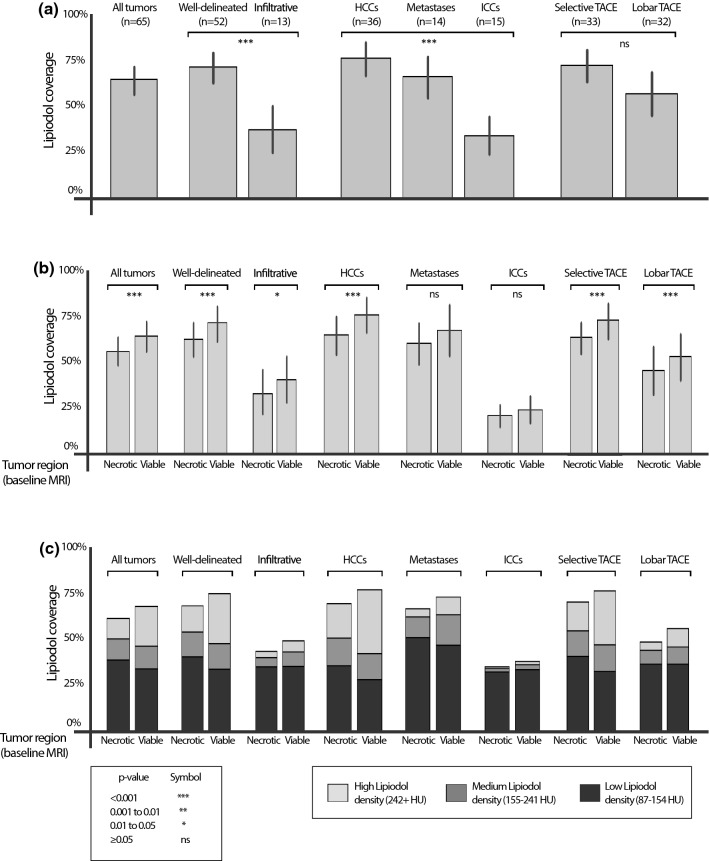
Lipiodol coverage. Lipiodol coverage was measured on 24 h CT, averaged over all tumors as well as various subgroups. *p* values are derived from statistical tests (Mann–Whitney U test, Kruskal–Wallis test, Wilcoxon signed-rank test) of subgroup differences in percent tumor coverage. Bars represent 90% confidence intervals. (**a**) The percent Lipiodol coverage of the whole tumor for various subgroups. (**b**) Necrotic and viable tumor coverage with Lipiodol. Tumor enhancement on baseline MRI was significantly associated with Lipiodol deposition on 24 h CT (*p* < 0.0001), with 8.22% ± 14.59 more Lipiodol coverage in viable areas than necrotic areas of the tumor on BL MRI (**c**) Lipiodol density distribution in viable versus necrotic areas. The fraction of low density Lipiodol in necrotic areas was significantly higher than in viable areas (difference of 8.10% ± 15.69, *p* = 0.0002), and not significantly different for mid density Lipiodol (*p* = 0.0933), whereas the fraction of high density Lipiodol in viable areas was significantly higher than in necrotic areas (17.21% ± 22.19, *p* < 0.0001). For exact *p* values as well as mean ± standard deviation (SD) for (**c**) see Supplemental Materials.

ETV on baseline MRI was significantly correlated with Lipiodol deposition on 24 h CT (*p* < 0.0001). Viable tumor areas on baseline MRI on average deposited 8.22% ± 14.59 (mean ± SD) more Lipiodol than necrotic areas (Fig. 4b). As illustrated in Fig. 4a, well-delineated tumors on average deposited more Lipiodol than infiltrative tumors [71.7% ± 27.9 vs. 37.5% ± 22.9 (mean ± SD), *p* = 0.0001]. Comparing Lipiodol deposition between HCC, ICC and metastases, HCCs deposited 76.9% ± 27.4 Lipiodol compared to ICC and Metastases, which only had an average Lipiodol deposition of 34.4% ± 19.9 and 66.7% ± 22.8 (mean ± SD), respectively (*p* < 0.0001). There was no significant difference in tumor Lipiodol deposition between patients treated with lobar and selective TACE (*p* = 0.1443).

Percent Lipiodol coverage at various densities was compared between baseline viable and necrotic tumor areas, as shown in Fig. 4c. The fraction of low density Lipiodol in necrotic areas was significantly higher than in viable areas [difference of 8.10% ± 15.69 (mean ± SD), *p* = 0.0002]. In contrast, the fraction of mid density Lipiodol did not differ significantly between necrotic and viable areas (*p* = 0.0933). The fraction of high density Lipiodol in viable areas was significantly higher than in in non-enhancing, presumably necrotic tumor tissue [7.21% ± 22.19 (mean ± SD), *p* < 0.0001].

### Lipiodol features and reduction in viable tumor tissue

**Figure 5:**
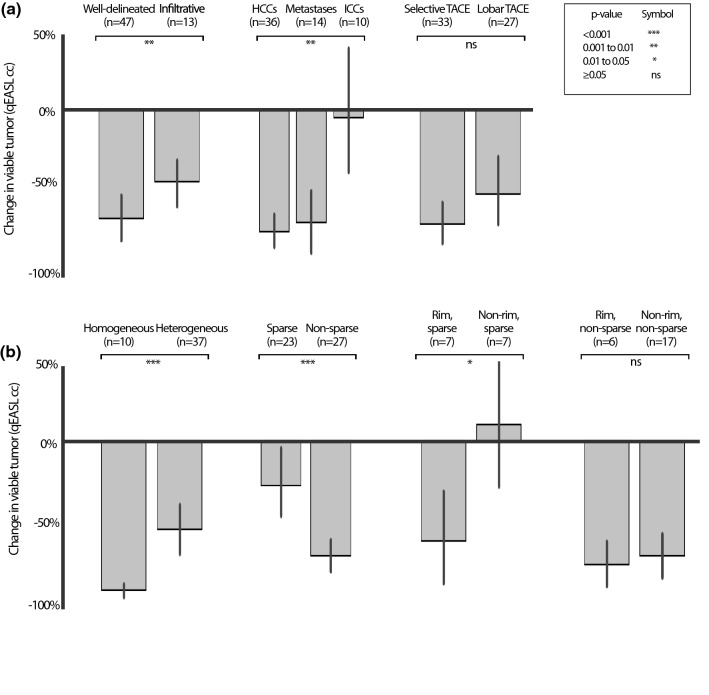
Percent change in viable tumor baseline MRI and follow up MRI. Reduction in viable tumor tissue between baseline MRI and follow-up MRI [%], compared between (**a**) tumor subgroups based on tumor growth (well-delineated vs. infiltrative), tumor entity (HCC vs. Metastases vs. ICC) and TACE approach (selective vs. lobar), as well as (**b**) patterns of Lipiodol deposition, where the comparisons with homogeneous or rim patterns were restricted to well-delineated tumors. Lesions were only included in the analysis if they had enhancing tumor tissue on baseline MRI. qEASL = quantitative European Association for the Study of the Liver (tumor response criteria).

Subgroup analysis was performed to compare overall reduction in viable tumor tissue, defined as change in ETV between baseline and follow-up MRI (Fig. 5). Well-delineated tumors had a significantly higher reduction in ETV than infiltrative tumors, with an average of − 63.0% ± 47.8 versus − 41.5% ± 25.1 (mean ± SD) (*p* = 0.0038). HCCs and metastases had a moderately higher reduction in ETV than ICC (− 70.7% ± 28.9, − 65.3% ± 36.9 and − 4.3% ± 62.2 (mean ± SD), *p* = 0.0064). There was no significant difference in ETV reduction between tumors treated with selective or lobar TACE approach (*p* = 0.4242).

Lipiodol deposition patterns identified on post-procedural CT showed significant differences in overall reduction in viable tumor tissue. Well-delineated tumors with homogeneous Lipiodol deposition on 24 h CT showed a higher reduction in ETV compared to tumors with heterogeneous deposition (− 92.9% ± 7.1 vs. − 54.9% ± 50.9 (mean ± SD), *p* = 0.0006). We found that tumors with sparse Lipiodol deposition on 24 h CT tended to have a smaller reduction in viable tumor tissue after cTACE than tumors with non-sparse heterogeneous deposition of Lipiodol (− 25.8% ± 66.6 vs. − 72.7% ± 27.2 (mean ± SD), *p* < 0.0001). Among well-delineated tumors with sparse Lipiodol deposition, tumors with rim deposition had a much higher reduction in ETV than tumors without rim deposition [− 62.0% ± 41.9 vs. + 10.5% ± 69.2 (mean ± SD), *p* = 0.0450]. However, there was no significant difference between non-sparse heterogeneous well-delineated tumors with and without rim deposition (*p* = 0.2888). Response rates of the analyzed lesions according to qEASL response criteria and stratified by different Lipiodol deposition pattern are demonstrated in Table 3.

**Table 3:** Response rates (qEASL) of lesions stratified by Lipiodol deposition patterns. Radiographic response was assessed according to the qEASL criteria. A responding lesion required the reduction of enhancing tumor tissue by at least 65%. Exact Fisher tests were used to determine significance between outcome groups. All lesions *p* < 0.001, well-delineated lesions *p* < 0.001 and Infiltrative lesions *p* = 0.077. A *p *value < 0.05 was considered statistically significant. qEASL, quantitative European Association for Study of the Liver.

	Responder (qEASL) n (%)	Non-responder (qEASL) n (%)
**All Lesions**	32 (49.2%)	33 (50.8%)
Sparse	5 (17.9%)	23 (82.1%)
Non-sparse	27 (73%)	10 (27%)
**Well-delineated lesions**		
Homogenous	10 (100%)	0 (0%)
Sparse	5 (26.3%)	14 (73.7%)
Non-sparse	25 (75.8%)	8 (24.2%)
Rim (all)	9 (64.3%)	5 (35.7%)
Rim non-sparse	4 (66.7%)	2 (33.3%)
Rim sparse	5 (62.5%)	3 (37.5%)
**Infiltrative lesions**		
Sparse	0 (0%)	9 (100%)
Non-sparse	2 (50%)	2 (50%)

**Figure 6:**
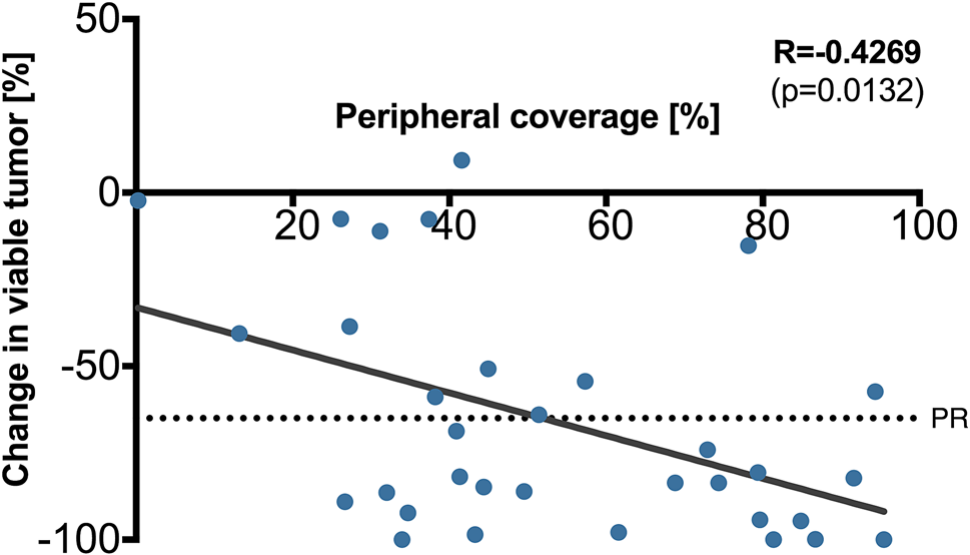
Peripheral Lipiodol coverage. Peripheral coverage with Lipiodol is associated with increased response. On average, a 1% increase in peripheral coverage contributes to a − 0.62% reduction in viable tumor tissue. PR = partial response (based on quantitative European Association for the Study of the Liver (qEASL) tumor response criteria).

On average, tumors treated with selective TACE had peripheral Lipiodol coverage of 53.5% ± 27.5 (mean ± SD). Peripheral coverage with Lipiodol was associated with increased reduction in viable tumor tissue. As illustrated in Fig. 6, an 1% increase in peripheral coverage on average contributed to a − 0.62% change in viable tumor volume (R = − 0.4269, *p* = 0.0132).

**Figure 7:**
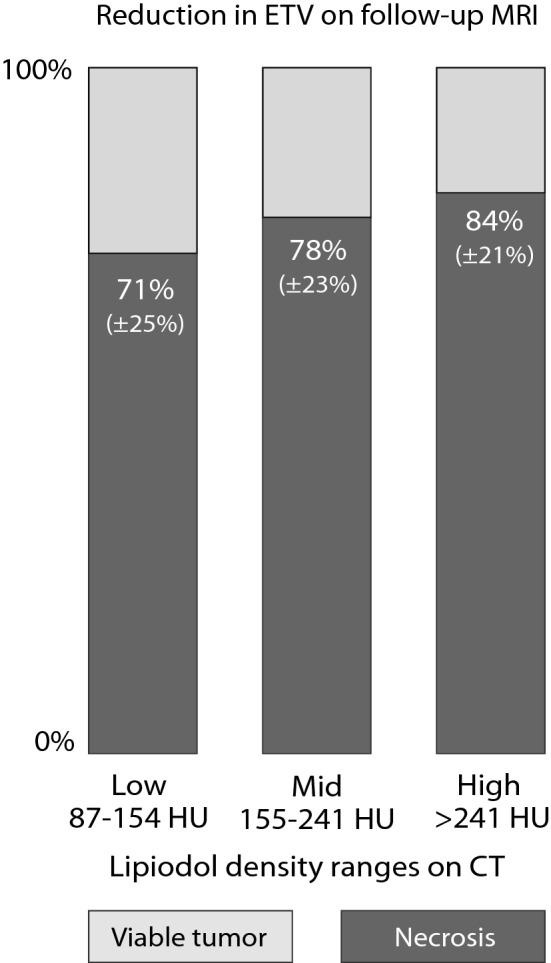
Percent reduction in viable tumor on follow-up MRI across tumors. Within viable tumor tissue on baseline MRI, areas of increasing Lipiodol density tend to become necrotic at a higher rate (*p* = 0.0008 for mid density Lipiodol vs. low density Lipiodol; *p* = 0.0051 for high density vs mid density Lipiodol). The percentages shown in the graph are averages of the individual percentages of voxels in each tumor that are viable or necrotic. Areas of the tumor are only included in the analysis if they contain at least 50 voxels.

Within viable tumor tissue on baseline MRI, areas with Lipiodol on 24 h CT tended to become necrotic at a higher rate on follow-up MRI than areas without Lipiodol (*p* = 0.00475). Higher concentrations of Lipiodol enhanced this effect: compared to areas with no Lipiodol deposition, the decrease of ETV in areas depositing low, medium and high Lipiodol within the same tumor was 0.87% ± 15.98 (*p* = 0.3393), 9.32% ± 22.20 (*p* = 0.0066) and 17.91% ± 23.42 (mean ± SD) (*p* = 0.0003) respectively. Across tumors, Lipiodol density is also significantly associated with the reduction of ETV within those regions, as illustrated in Fig. 7. Areas of mid density Lipiodol deposition had significantly higher reduction of ETV than areas of low density Lipiodol (*p* = 0.0008), and areas of high density Lipiodol had higher reduction of ETV than areas of mid density Lipiodol (*p* = 0.0051).

## Discussion

This study demonstrates that an automated volumetric characterization of Lipiodol deposits on 24 h post-cTACE CT is feasible and shows how patterns and densities of Lipiodol can be practically used to predict reduction in enhancing tumor tissue and thus response on follow-up imaging. Additionally, we found that Lipiodol deposition in target lesions can be predicted pre-procedurally by assessing tumor enhancement on baseline MRI.

The role of Lipiodol as potential imaging biomarker has been previously suggested with evidence from both retrospective and prospective data. Previous studies indicate that Lipiodol coverage on CT is correlated with subsequent necrosis on follow-up imaging and histopathology^10–13,28,32,33^. Unlike particulate-form embolic agents that usually fail to effectively penetrate the small intra-tumoral vasculature and tissue, Lipiodol, due to its oily nature, enters the tumor more readily and is retained within the tissue in a likely tumor-specific manner. Theoretically, the quantification of density and spatial distribution of Lipiodol on 24 h CT could enable the assessment of drug delivery success, identification of undertreated areas and prediction of response^10,11,22,32^. Our methods translate this hypothesis into practice and provide proof-of-concept for density and pattern-based prediction of tumor tissue devascularization in areas of Lipiodol deposition throughout several tumor entities. Tumor areas depositing higher concentrations of Lipiodol as well as tumors with homogenous and non-sparse deposition patterns show higher rates of ETV reduction and could be predictive of response.

Additionally, our findings indicate that tumors with peritumoral deposition of Lipiodol have a higher reduction of viable tumor tissue after cTACE, potentially due to the unique blood supply and capillary pathoanatomy of hepatic malignancies. It has been shown that these tumors are preferentially supplied by the high-pressure arterial system, which generates high resistance at the tumor margins preventing portal venous flow^9^. Embolic impairment of the arterial blood supply alone (e.g. by drug-eluting beads) therefore still allows for portal venous blood flow. Lipiodol that has drained through arterio-portal shunts at the tumor margin into the immediate peritumoral periphery or as rim deposition may thus promote increased ischemia.

Our data also validated the role of arterial phase hyperenhancement on pre-procedural MRI as a predictor for Lipiodol deposition. Arterial-phase enhancement captures well-perfused areas that increase Lipiodol uptake through the hepatic artery^9^. Accordingly, hypervascularized HCCs showed the greatest Lipiodol deposition among analyzed entities and a very high fraction of high density Lipiodol in viable areas^4^. Also, well-delineated tumors took up more Lipiodol than infiltrative tumors, which may be explained by the presence of a capsule and lack of central necrosis due to hypoperfusion.

As for the practical value of automated characterization of Lipiodol deposits, previous studies proposed semi-automatic thresholding techniques that are applied separately for each patient, without leveraging the standardized attenuation measure on CT provided by the HU scale. Our study is a technical innovation study that proposes to apply fully automated image analysis to characterize Lipiodol deposition, allowing for the future development of scoring systems that make use of both tumor coverage and other deposition characteristics to predict response in a robust and time-efficient manner.

After validation with histopathology or long-term radiological follow-up imaging, the automated techniques for analysis of Lipiodol coverage, patterns and density presented here could possibly have several clinical implications: In terms of patient selection, arterially hyperenhancing lesions on baseline imaging represent a phenotype that is more likely to respond to therapy. Tumors showing high density, homogenous, non-sparse and peripheral deposition of Lipiodol on 24 h CT are more likely to devascularize. For patients with poor Lipiodol deposition or unfavorable distribution patterns on 24 h CT, additional treatments such as targeted thermal ablation of insufficiently covered tumor portions could add significant value and improve complete response rates without the need to delay for MRI-based tumor response assessment. In this context, recent studies demonstrated the benefits of a short time interval when combining cTACE with ablation^34^. Our quantitative techniques therefore have the potential to improve and standardize decision making when combining TACE with ablation or other adjuvant therapies.

While intra-procedural cone-beam CT (CBCT) imaging is commonly applied in a non-quantitative fashion to visualize Lipiodol deposition during and immediately after cTACE, the acquired data lack calibration of signal and, in the absence of measurable HU, prohibit true measurement of density and pattern. Therefore, this technical limitation continues to necessitate the acquisition of conventional CT images 24 h post-cTACE in order to be able to use Lipiodol as a quantifiable imaging biomarker^19^. Future optimization efforts should focus on introducing reliable calibration of CBCT in order to allow for automated intra-operative characterization of Lipiodol deposition in real time.

The study has some limitations. Data was collected from a heterogeneous cohort of different primary and secondary liver tumors, with varying TACE protocols, MR and CT scanners, and drug cocktails administered. However, these reflect real-world clinical practice, and all included patients were enrolled in prospective clinical trials with similar protocols, differing merely in their observed endpoints. Focusing on our technical innovation as proof-of-concept, the tumor heterogeneity improves the robustness of the model and demonstrates that this method may be applicable to a wide range of liver tumors. Due to possible imprecision in the segmentation and registration, solely lesions ≥ 1 cm were included and subregions were only analyzed if they contained ≥ 50 voxels. Additionally, our follow-up period was rather short and histopathological proof was not available to validate necrosis, which was assumed to be present in devascularized tissue on follow-up MRI. However, such findings on contrast-enhanced MRI are known to be strongly correlated with necrosis on pathology after TACE, and have been used in clinical trials and treatment management as a key surrogate marker^25,35^.

In summary, this study established quantitative automated threshold-based techniques to characterize Lipiodol deposition patterns and densities on post-procedural CT to be used as imaging biomarkers for therapeutic efficacy of cTACE.

## Supplementary information

Supplementary Information.

## Data Availability

The authors guarantee availability of materials and data without any restrictions.

## Abbreviations

BCLC: Barcelona clinic liver cancer
CT: Computed tomography
cTACE: Conventional transarterial chemoembolization
ETV: Enhancing tumor volume
HCC: Hepatocellular carcinoma
IAT: Intraarterial therapy
ICC: Intrahepatic cholangiocarcinoma
KW: Kruskal–Wallis test
LiRADS: Liver imaging reporting and data system
MRI: Magnetic resonance imaging
MWU: Mann–Whitney U test
VOI: Volume of interest
WSR: Wilcoxon signed-rank test
