# Supplementary material for: Automated feature quantification of Lipiodol as imaging biomarker to predict therapeutic efficacy of conventional transarterial chemoembolization of liver cancer
Source: Sci Rep. 2020 Oct 22;10:18026. doi: 10.1038/s41598-020-75120-7 (PMC7582153; doi:10.1038/s41598-020-75120-7)
Supplement: Supplementary file 1 — Supplementary Information. [file 41598_2020_75120_MOESM1_ESM.pdf]

Title: Automated Feature Quantification of Lipiodol as an Imaging Biomarker to Predict Therapeutic Efficacy of Conventional Transarterial Chemoembolization of Liver Cancer

Authors: Sophie Stark[1,2,4], Clinton Wang[1], Lynn Jeanette Savic, MD[1,2], Brian Letzen, MD[1], Isabel Schobert[1,2], Milena Miszczuk, MD[2], Nikitha Murali[1], Paula Oestmann[1,2,5], Bernhard Gebauer, MD[2], MingDe Lin, PhD[1], James Duncan PhD[1,3], Todd Schlachter, MD[1], Julius Chapiro, MD, PhD[1]

[1] Department of Radiology and Biomedical Imaging, Yale School of Medicine, 333 Cedar Street, New Haven, CT 06510
[2] Charité - Universitätsmedizin Berlin, corporate member of Freie Universität Berlin, Humboldt-Universität, and Berlin Institute of Health, 10117 Berlin, Germany, Institute of Radiology
[3] Department of Biomedical Engineering, Yale School of Engineering and Applied Science, New Haven, CT 06520
[4] Faculty of Medicine, Albert-Ludwigs-University Freiburg, Germany
[5] Faculty of Medicine, Heinrich-Heine-University Düsseldorf, Germany

Supplemental Materials:

Materials and Methods:

Statistical Analysis

Statistical tests were performed using GraphPad Prism 7.0 (GraphPad Software, La Jolla California USA, www.graphpad.com). A p-value <0.05 was considered statistically significant. Total Lipiodol coverage was correlated between different groups of tumors, using the Mann-Whitney U (MWU) test for comparing two groups and the Kruskal-Wallis (KW) test for comparing three or more groups. Specifically, Lipiodol coverage was correlated with tumor growth (well-delineated or infiltrative) and TACE approach (selective or lobar) using the MWU test, and was correlated with tumor entity (HCC, ICC, or Metastases) using the KW test. The Wilcoxon signed-rank (WSR) test was used to analyze the difference between Lipiodol coverage in viable and necrotic tumor regions. The KW and MWU tests were also used to determine whether this difference between viable coverage and necrotic coverage varied between the tumor groups mentioned previously. This difference between viable and necrotic coverage was then evaluated for specific densities of Lipiodol using the WSR test. Average overall tumor response was compared between tumor categories based on tumor entity, tumor growth, TACE approach, and homogeneous, sparse, and rimmed Lipiodol deposition patterns, using MWU and KW tests. Peripheral Lipiodol deposition was correlated with overall tumor response using linear regression and Spearman's rank-order correlation, restricted to lesions that underwent selective TACE. This restriction was used because peripheral Lipiodol deposition may either reflect off-target Lipiodol that accumulates in healthy liver parenchyma or Lipiodol that drained into vessels surrounding the tumor. Whereas almost all lesions treated with lobar TACE had some peripheral off-target deposition, lesions that underwent selective TACE were more likely to exhibit the latter effect, making it a

potentially useful biomarker. Additionally, the response of viable tumor tissue on baseline MRI was compared between areas of the tumor with no Lipiodol deposition and areas of the tumor with Lipiodol of specific density, using the WSR test.

Supplementary Figures:

a)

Category	N	Any Lipiodol	Low Lipiodol	Mid Lipiodol	High Lipiodol
**All Tumors**	65	**<0.0001**	***0.0002***	*0.0933*	**<0.0001**
**Well-delineated**	52	**0.0011**	***<0.0001***	*0.2571*	**<0.0001**
**Infiltrative**	13	**0.0266**	0.5417	*0.0803*	0.0830
**HCC**	36	**0.0002**	***0.0003***	*0.7613*	**<0.0001**
**ICC**	15	*0.4316*	0.5566	*0.2969*	*>0.9999*
**Metastases**	14	0.1353	*0.0906*	***0.0266***	**0.0068**
**Selective TACE**	33	**0.0088**	***0.0012***	*0.1821*	**0.0005**
**Lobar TACE**	32	**0.0009**	*0.0890*	*0.3294*	**0.0007**

b)

Category	N	Any Lipiodol Mean ± SD (%)		Low Lipiodol Mean ± SD (%)		Mid Lipiodol Mean ± SD (%)		High Lipiodol Mean ± SD (%)	
		viable	necrotic	viable	necrotic	viable	necrotic	viable	necrotic
**All Tumors**	**65**	66 ± 31.9	57.6 ± 29.6	25.0 ± 14.5	32.9 ± 18.9	20.7 ± 16.1	16.9 ± 17.2	20.3 ± 26.5	7.8 ± 13.1
**Well-delineated**	**52**	73.7 ± 30.2	64.5 ± 28.0	24.4 ± 15.0	35.2 ± 19.0	23.1 ± 16.5	20.4 ± 17.9	26.1 ± 28.0	9.7 ± 14.3
**Infiltrative**	**13**	40.9 ± 23.3	33.3 ± 21.9	25.4 ± 12.6	24.2 ± 15.7	11.5 ± 10.0	7.1 ± 7.6	4.0 ± 4.2	2.0 ± 3.1
**HCC**	**36**	78.0 ± 27.4	67.9 ± 29.4	21.0 ± 13.7	32.5 ± 18.8	23.9 ± 14.3	23.1 ± 18.5	33.1 ± 28.5	12.4 ± 15.3
**ICC**	**15**	24.2 ± 12.0	33.2 ± 20.7	19.3 ± 6.9	18.2 ± 7.1	3.9 ± 5.3	2.3 ± 2.8	1.0 ± 1.9	0.6 ± 1.2
**Metastases**	**14**	67.5 ± 26.5	60.4 ± 21.9	37.8 ± 12.7	44.2 ± 17.3	24.1 ± 18.1	14.0 ± 11.0	5.6 ± 5.2	2.2 ± 3.0
**Selective TACE**	**33**	73.3 ± 28.0	63.8 ± 24.5	25.9 ± 14.7	34.1 ± 16.5	22.3 ± 14.4	18.6 ± 13.9	25.1 ± 27.4	11.1 ± 16.4
**Lobar TACE**	**32**	58.3 ± 34.3	52.6 ± 33.1	23.1 ± 14.2	31.3 ± 21.3	18.5 ± 17.7	16.2 ± 20.4	16.7 ± 24.6	4.2 ± 6.4

Supplementary Table 1: Additional information to Figure 3c: Comparison of percent Lipiodol coverage for baseline (BL) viable vs BL necrotic areas, as well as comparison of percent Lipiodol coverage with low, mid,

high density between viable and necrotic areas of the tumor. a) P-values are derived from Wilcoxon signed-rank test and refer to the difference in Lipiodol deposition between viable and necrotic areas, i.e. is there a difference between the percent of any/low/mid/high density Lipiodol that can be found in viable tissue (by volume) vs. the percent of any/low/mid/high density Lipiodol found in necrotic tissue. Areas were only included in the analysis if they had at least 50 voxels of a certain Lipiodol density. Italic font indicates that Lipiodol of a certain density is higher in necrotic tumor areas, otherwise Lipiodol of that density is higher in viable tumor areas. A p-value <0.05 was considered statistically significant as indicated in bold font. b) Mean and standard deviation (SD) for Figure 3c are shown in percent.
